# Enzymatical and microbial degradation of cyclic dipeptides (diketopiperazines)

**DOI:** 10.1186/2191-0855-3-51

**Published:** 2013-08-30

**Authors:** Mareike Perzborn, Christoph Syldatk, Jens Rudat

**Affiliations:** 1 Karlsruhe Institute of Technology, Institute of Process Engineering in Life Sciences, Section II: Technical Biology, Engler-Bunte-Ring 1, 76131 Karlsruhe, Germany

**Keywords:** Diketopiperazines, Cyclic dipeptides, Peptide bond, Degradation, Hydrolysis, Biotransformation, Cyclic amidases, Peptidases

## Abstract

Diketopiperazines (DKPs) are cyclic dipeptides, representing an abundant class of biologically active natural compounds. Despite their widespread occurrence in nature, little is known about their degradation. In this study, the enzymatical and microbial cleavage of DKPs was investigated. Peptidase catalyzed hydrolysis of certain DKPs was formerly reported, but could not be confirmed in this study. While testing additional peptidases and DKPs no degradation was detected, indicating peptidase stability of the peptide bond in cyclic dipeptides. Besides confirmation of the reported degradation of cyclo(l-Asp-l-Phe) by *Paenibacillus chibensis* (DSM 329) and *Streptomyces flavovirens* (DSM 40062), cleavage of cyclo(l-Asp-l-Asp) by DSM 329 was detected. Other DKPs were not hydrolyzed by both strains, demonstrating high substrate specificity. The degradation of cyclo(l-Asp-l-Phe) by DSM 40062 was shown to be inducible. Three strains, which are able to hydrolyze hydantoins and dihydropyrimidines, were identified for the degradation of DKPs: *Leifsonia* sp. K3 (DSM 27212) and *Bacillus* sp. A16 (DSM 25052) cleaved cyclo(dl-Ala-dl-Ala) and cyclo(l-Gly-l-Phe), and *Rhizobium* sp. NA04-01 (DSM 24917) degraded cyclo(l-Asp-l-Phe), cyclo(l-Gly-l-Phe) and cyclo(l-Asp-l-Asp). The first enantioselective cleavage of cyclo(dl-Ala-dl-Ala) was detected with the newly isolated strains *Paenibacillus* sp. 32A (DSM 27214) and *Microbacterium* sp. 40A (DSM 27211). Cyclo(l-Ala-d-Ala) and cyclo(l-Ala-l-Ala) were completely degraded, whereas the enantiomer cyclo(d-Ala-d-Ala) was not attacked. Altogether, five bacterial strains were newly identified for the cleavage of DKPs. These bacteria may be of value for industrial purposes, such as degradation of undesirable DKPs in food and drugs and production of (enantiopure) dipeptides and amino acids.

## Introduction

Diketopiperazines (DKPs) are the smallest possible cyclic peptides composed of two α-amino acids. They are abundant natural compounds produced by a variety of organisms (Borthwick 2012): bacteria like *Bacillus subtilis* (Elkahoui et al. 2012) and *Streptomyces* sp. (Johnson et al. 1951), fungi, e.g., *Alternaria alternata* (Stierle et al. 1988) and *Penicillium* sp. (Du et al. 2009), and sponges, for example, *Dysidea fragilis* (Su et al. 1993). They were identified in mammals, e.g., in rat and monkey brains, human central nervous system, gastrointestinal tract and blood (Prasad 1988). Besides the widespread DKP biosynthesis, they occur as chemical degradation products in e.g., roasted coffee (Ginz and Engelhardt 2000), stewed beef (Chen et al. 2009) and beer (Gautschi et al. 1997). The identified DKPs were described to cause a bitter taste in these foods. Furthermore, DKPs are formed as decomposition products of drugs by cyclization, e.g., of the aminopenicillin antibiotic amoxicillin (Lamm et al. 2009). Amoxicillin-2,5-diketopiperazine, the chemically stable form of amoxicillin was detected in wastewater samples, and may elicit allergic reactions in human consumers of water and food of animal origin (Lamm et al. 2009). Identification of DKP cleaving enzymes or microorganisms could be important for the degradation of these interfering by-products in food and drug industry.

DKPs exhibit diverse bioactivities including antibacterial (Fdhila et al. 2003), antifungal (Ström et al. 2002) and antiviral activity (Sinha et al. 2004), as well as cytotoxicity (McCleland et al. 2004) and phytotoxicity (Stierle et al. 1988). Moreover, DKPs were shown to act as quorum sensing molecules (Ryan and Dow 2008). Cyclo(l-Pro-l-Tyr), used in this study, was identified in culture supernatant of e.g., *Pseudomonas aeruginosa*, and was shown to activate an *N*-acylhomoserine lactone biosensor (Holden et al. 1999).

Little is known about the biodegradation of these molecules. There are only few studies describing the enzymatical or microbial hydrolysis of DKPs.

Some peptidases (also termed proteases, EC 3.4.X.X) are reported to cleave the peptide bonds in DKPs. However, some of the results are inconsistent and to the best of our knowledge the last report about this topic was published in 1940.

The first cleavage of a peptide bond in DKPs was reported for cyclo(Asp-Asp), cyclo(Gly-Asp) and cyclo(Gly-Glu) by trypsin (Matsui 1933). However, hydrolysis of cyclo(Gly-Asp) and cyclo(Gly-Glu) by trypsin was disproved afterwards (Akabori and Takase 1936). Cleavage of cyclo(Asp-Asp) and cyclo(Gly-Glu) by papain was demonstrated by Shibata and Tazawa (1936), but the last could not be confirmed by Akabori and Takase (1936) and Itibakase (1940). Overall, cleavage of the peptide bond in DKPs by peptidases is poorly studied.

Besides the enzymatical cleavage of DKPs, the microbial hydrolysis is reported for the following bacteria. *Bacillus* sp. No. 106 degraded cyclo(Gly-Gly) (Muro et al. 1985). *Arthrobacter* sp. 1-3-1 and a coryneform rod bacterium T-1-3-Y hydrolyzed different tyrosine- and glycine-containing DKPs (Kanzaki et al. 1997). *Agrobacterium radiobacter* NM 5-3 cleaved glycine-containing DKPs (Kanzaki et al. 2000). The biodegradation of cyclo(Gly-Leu) was reported to proceed in two steps with two distinct enzymes. A cyclo(Gly-Leu) hydrolase is responsible for the DKP hydrolysis, and a dipeptidase cleaves the formed dipeptides to the corresponding amino acids (Kanzaki et al. 2000). It is not clear which enzymes are responsible for the catalysis of the first reaction step.

To the best of our knowledge, none of the named strains is deposited in an international strain collection.

Two applications for DKP hydrolysis concerning the sweetener aspartame (*N*-(l-α-aspartyl-l-phenylalanine methyl ester) are described. First, under alkaline conditions cyclo(l-Asp-l-Phe) is formed as cyclization product of aspartame and has a bitter taste in contrast to the sweet taste of aspartame. *Bacillus circulans* was identified for the hydrolysis of cyclo(l-Asp-l-Phe) and can be used for degradation of this interfering by-product (Pantaleone et al. 1998). Second, for establishing a novel process for the synthesis of aspartame, Yokozeki et al. (1990) screened for microorganisms hydrolyzing cyclo(l-Asp-l-Phe) to the corresponding linear dipeptide l-Asp-l-Phe, which can be converted to aspartame by methyl esterification. Two of the isolated strains, *Bacillus circulans* ATCC 9966 (equates *Paenibacillus chibensis* DSM 329) and *Streptomyces flavovirens* IFO 3197 (DSM 40062), were used in our study to investigate DKP hydrolysis.

**Figure 1:**
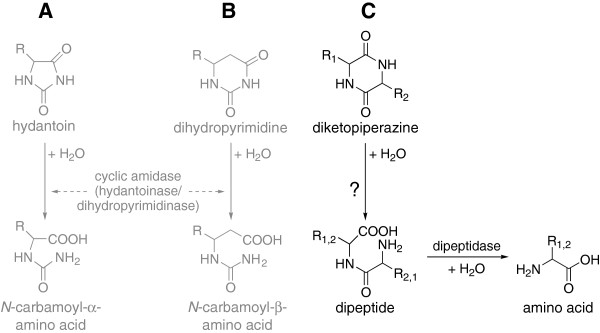
**Enzymatical hydrolysis of different cyclic amides. (A)** Hydantoins are cleaved to *N*-carbamoyl-α-amino acids. **(B)** Dihydropyrimidine derivatives are converted to *N*-carbamoyl-β-amino acids. **(C)** Potential biodegradation way of DKPs: hydrolysis of DKPs would lead to the corresponding linear dipeptides by unknown enzymes (investigated in this work), and the formed dipeptides could be hydrolyzed to the corresponding amino acids by dipeptidases.

The hydantoinase process is used for the production of enantiopure α-amino acids in industrial scale (Altenbuchner et al. 2001). At first, hydantoins are hydrolyzed to the corresponding *N*-carbamoyl-α-amino acids by hydantoinases (Figure 1A). The applicability of this process for the hydrolysis of dihydropyrimidines to the corresponding *N*-carbamoyl-β-amino acid (Figure 1B) for the production of β-amino acids was tested by Engel et al. (2012). It was shown that the same cyclic amidases are able to cleave hydantoins (Dürr et al. 2008) and dihydropyrimidines (Engel et al. 2012). Moreover, various bacterial strains were identified for the simultaneous hydrolysis of hydantoins and dihydropyrimidines (Dürr et al. 2006). Due to the structural similarity between hydantoins, dihydropyrimidines and DKPs, strains with cyclic amidase activity of the in-house strain collection were tested for DKP hydrolysis. This reaction would lead to the corresponding linear dipeptide, which can be cleaved by dipeptidases to α-amino acids (Figure 1C).

We investigated the enzymatical and microbial degradation of DKPs composed of proteinogenic and non-proteinogenic amino acids by four different approaches: (1) cleavage of the peptide bond in DKPs by peptidases, (2) substrate specificity and enzyme induction with *Paenibacillus chibensis* (DSM 329) and *Streptomyces flavovirens* (DSM 40062), which are described for the hydrolysis of cyclo(l-Asp-l-Phe), (3) degradation of DKPs with strains known for the hydrolysis of other cyclic amides (hydantoins and dihydropyrimidines), and (4) identification of novel isolates with enantioselective activity towards one racemic DKP.

## Materials and methods

### Chemicals and reagents

DKPs cyclo(l-Ala-l-Ala), cyclo(l-Pro-l-Tyr), cyclo(l-Asp-l-Asp) and cyclo(Gly-l-Phe) were purchased from Bachem Holding (Bubendorf, Switzerland), cyclo(dl-Ala-dl-Ala) and cyclo(l-Asp-l-Phe) were obtained from Sigma-Aldrich (St. Louis, USA), cyclo(Gly-Gly) was received from TCI Europe N.V. (Zwyndrecht, Belgium) and cyclo(l-Arg-l-Arg) acetate salt, cyclo(l-Lys-l-Lys) acetate salt, (*S*)-3-benzyl-1,4-dimethyl-2,5-diketopiperazine, 3-benzyl-3-methyl-2,5-diketopiperazine and 1,3-dimethyl-2,5-diketopiperazine were provided by Taros Chemicals Corporation (Dortmund, Germany). Dihydrouracil, NaCl and MgSO_4_ × 7 H_2_O were purchased from Sigma-Aldrich (St. Louis, USA). Methanol ROTISOLV® HPLC Gradient Grade (MeOH), peptone from casein, K_2_HPO_4_, KH_2_PO_4_, Na_2_HPO_4_ × 2 H_2_O, NaH_2_PO_4_ × 2 H_2_O, sodium acetate trihydrate, TRIS, EDTA, 99% acetic acid, NaOH, ampicillin sodium salt, l-rhamnose monohydrate, agarose NEEO Ultra and ethidium bromide were supplied by Carl Roth Corporation (Karlsruhe, Germany). Tryptone and yeast extract were obtained from Becton, Dickinson and Company (Franklin Lakes, USA).

### Peptidases

Trypsin (5,000 USP-U/mg), pepsin (≥0.5 E/mg), papain (>30,000 USP-U/mg) and chymotrypsin (>1,000 USP-E/mg) were received from Carl Roth Corporation (Karlsruhe, Germany). Ficin FSM 200 and bromelain Br 400 were purchased from Enzybel International SA (Villers-le-Bouillet, Belgium). Alcalase (subtilisin), savinase (subtilisin), everlase (subtilisin), Esperase (subtilisin), Protex 6L, Protex 30L, Protex 40L, Protex 51 FP and Protex 89L were obtained as immobilized enzymes as part of the Immozyme™ protease Kit (Chiral Vision, Leiden, Netherlands).

### Bacterial strains

*Paenibacillus chibensis* (DSM 329) and *Streptomyces flavovirens* (DSM 40062) were purchased from German Collection of Microorganisms and Cell Cultures (DSMZ, Braunschweig, Germany).

The following 34 strains hydrolyzing cyclic amides (hydantoins and dihydropyrimidines) of the in-house strain collection were tested for DKP hydrolysis: *Rhizobium* sp. NA04-01 (DSM 24917) isolated and investigated by Engel et al. (2012); *Leifsonia* sp. K3 (DSM 27212), *Burkholderia* sp. M3, *Flavobacterium* sp. F8, *Streptomyces* sp. I20, 4 *Bacillus* species (A16 (DSM 25052), F18, G18, H20), 3 *Pseudomonas* species (G7, M18, L9), 4 *Ochrobactrum* species (C15, D24, F21 (DSM 25042), I21) and 2 *Arthrobacter* species (E7 (DSM 24883), K20) isolated by Dürr et al. (2006); 5 unclassified strains (725, 157, 158, 222, 225), 2 *Aminobacter* species (728 (DSM 24754), 735 (DSM 24755)) and *Mesorhizobium* sp. (731) isolated from marine sediments and kindly provided by Dr. A. Puñal (Engel et al. 2012); 2 *Arthrobacter polychromogenes* (DSM 20136, DSM 342), *Arthrobacter aurescens* (DSM 20116), *Arthrobacter nicotinovorans* (DSM 420), *Arthrobacter citreus* (DSM 20133) and *Arthrobacter sulfureus* (DSM 20167) provided by DSMZ (Braunschweig, Germany) and two strains with recombinant d-hydantoinases (*Escherichia coli* (*E. coli*) K12 JM109 with pJOE5702.1 and with pJOE5704.1) constructed by Dürr et al. (2008). The wild type strain *E. coli* K12 JM109 (New England Biolabs, Ipswich, USA) was used as reference strain.

*Paenibacillus* sp. 32A (DSM 27214) and *Microbacterium* sp. 40A (DSM 27211) were isolated from biotransformation experiments with 5 mM cyclo(dl-Ala-dl-Ala) and crude extract of DSM 329 and DSM 40062 in 50 mM sodium phosphate buffer (pH 7.5). After shaking at 30°C and 1,400 rpm for 9 d spontaneous activity was detected in these samples. Thus, samples were plated out on LB agar plates and incubated at 30°C for 6 d. Two colonies were isolated and the novel isolates were classified and tested for enantioselective hydrolysis of cyclo(dl-Ala-dl-Ala).

### 16S rRNA gene sequencing and strain classification

The genomic DNA of bacterial isolates was isolated from overnight cultures with the FastDNA® Spin Kit for Soil (MP Biomedicals, Santa Ana, USA) according to the manufacturer’s instructions. The polymerase chain reaction (PCR) to amplify the 16S rRNA gene was performed with genomic DNA, the universal bacterial primer set 27f (5'-AGAGTTTGATC(AC)TGGCTCAG-3') and 1385r (5'-CGGTGTGT(AG)CAAGGCCC-3') (Lane 1991), DNA-free water (Molzym, Bremen, Germany), dNTP Mix (GE Healthcare, Chalfont St. Giles, UK), PCR buffer and HotStarTaq DNA Polymerase (Qiagen, Hilden, Germany) following the manufacturer’s instructions. A mastercycler gradient (Eppendorf, Hamburg, Germany) was used and the conditions were as follows: initial denaturation (15 min, 95°C), followed by 30 cycles of denaturation (1 min, 94°C), annealing (1 min, 55°C), elongation (1.5 min, 72°C), and one final elongation step (10 min, 72°C). Quality and quantity of PCR products were controlled by agarose gel electrophoresis using 1% agarose in TAE buffer. Gels were stained with ethidium bromide and analyzed under UV light.

The PCR products were sequenced by GATC Biotech corporation (Konstanz, Germany) and identified by comparison with the GenBank® database (settings: nucleotide collection (nr/nt), exclude uncultured/environmental sample sequences, default megablast algorithm parameters).

### Cultivation

A bacterial colony was inoculated in 5 ml medium and strains were grown overnight at 30°C and shaking at 140 rpm. These precultures were used to inoculate fresh medium (10-100 ml) resulting in a starting OD_600nm_ of 0.05 (except for *Streptomyces flavovirens* (DSM 40062), for which OD_600nm_ cannot be determined, because this bacteria grows as a complex mycelium). Strains were cultivated at 30°C and 120 rpm for 24 h, and OD_600nm_ was measured with a spectrophotometer (Ultrospec 1100 *pro* UV/VIS, GE Healthcare, Chalfont St. Giles, UK).

*Paenibacillus chibensis* (DSM 329) and *Streptomyces flavovirens* (DSM 40062) were cultivated in modified complex medium (10.0 g/L peptone, 10.0 g/L yeast extract, 3.0 g/L K_2_HPO_4_, 1.0 g/L KH_2_PO_4_, 0.5 g/L MgSO_4_ × 7 H_2_O, adjusted to pH 7.2) (Yokozeki et al. 1990). To investigate the induction of enzyme activity, DSM 329 and DSM 40062 were cultivated in this complex medium supplemented with DKPs (1.25 g/L cyclo(Gly-Gly), 1.25 g/L, cyclo(dl-Ala-dl-Ala), 1.25 g/L cyclo(l-Asp-l-Phe)) and without DKPs. Afterwards, hydrolysis of 10 mM cyclo(l-Asp-l-Phe) was determined with resting cells. In addition, DSM 40062 was cultivated in medium supplemented with 0, 0.25, 0.5 and 1 mg/ml cyclo(l-Asp-l-Phe), and the influence of the DKP concentration on enzyme induction was tested with crude extract. Based on the results, DSM 40062 was cultivated with 1 mM cyclo(l-Asp-l-Phe) added to the medium for further experiments.

Strains of the in-house strain collection, exhibiting cyclic amidase activity, were cultivated in LB medium modified according to Bertani (1951) (10.0 g/L tryptone, 5.0 g/L yeast extract, 10.0 g/L NaCl, adjusted to pH 7.0). For cultivation of the two recombinant *E. coli* strains 0.1 g/L ampicillin was added to the medium. After reaching an OD_600nm_ of approximately 0.5, expression of recombinant d-hydantoinases was induced with 0.2% rhamnose.

The two novel isolates *Paenibacillus* sp. 32A (DSM 27214) and *Microbacterium* sp. 40A (DSM 27211) were cultivated in LB medium supplemented with 5 mM cyclo(dl-Ala-dl-Ala).

### Preparation of biocatalysts

For biotransformation experiments enzymes, resting cells or crude extract were used as biocatalyst.

Peptidases were mixed with 50 mM sodium phosphate buffer (pH 7.5), except for pepsin which was dissolved in 50 mM sodium acetate buffer (pH 3.1).

For preparation of resting cells and crude extract the cultivated cells were harvested by centrifugation (4,816 × g, 30 min, 4°C). The supernatant was discarded and the cells were washed three times with 50 mM sodium phosphate buffer (pH 7.5) followed by centrifugation. Resting cells were obtained by resuspension of the cell pellet in the same buffer. The bacterial cell dry mass was determined in triplicate by pipetting 1 ml washed cells in predried reaction tubes (60°C, 24 h), centrifugation (24,725 × g, 20 min, 4°C), drying of the cell pellets (60°C, 24 h) and gravimetrical quantification. Crude extract was prepared by sonication of washed cells for 8.5 min using alternate intervals of 30 sec pulsation on and 30 sec pulsation off and 35% amplitude (Sonopuls HD 3100 with ultrasonic probe MS 72, Bandelin electronic Corporation, Berlin, Germany). Disrupted cells were centrifuged (4,816 × g, 30 min, 4°C) and the protein concentration of the crude extract was determined in triplicate with the Bio-Rad Protein Assay (Bio-Rad Laboratories, Hercules, USA) according to the manufacturer’s instructions.

### Biotransformation

Biotransformation reactions were performed with peptidases, resting cells or crude extract mixed with 5 mM substrate (DKPs or dihydrouracil) dissolved in 50 mM sodium phosphate buffer (pH 7.5), except of 1 mM 3-benzyl-3-methyl-2,5-diketopiperazine.

Experiments were carried out in a Thermomixer (Eppendorf, Hamburg, Germany) at 30°C and shaking at 1,400 rpm. At different points of time, samples were taken and reactions were stopped by centrifugation (24,725 × g, 20 min, 4°C) of resting cells and immobilized peptidases, or by heat treatment (1,000 rpm, 90°C, 10 min) followed by centrifugation (24,725 × g, 20 min, 4°C) of crude extract and enzymes. The supernatant was diluted 1:2 with 50 mM sodium phosphate buffer (pH 7.5) and analyzed by HPLC analysis.

For biotransformations with peptidases the following conditions were used: enzyme concentration of 5 g/L for trypsin, pepsin and papain, 20 g/L for ficin, bromelain and chymotrypsin, and 10 g/L for the immobilized enzymes (Immozyme™ protease Kit); reaction temperature was 37°C for trypsin, 30°C for pepsin and papain, 40°C for ficin, bromelain and chymotrypsin, and 25°C for the immobilized enzymes. All reactions were carried out at 1,000 rpm, the reaction time was between 24 and 67 h, and samples were taken at four points of time. Reactions with pepsin were carried out at pH 2.4 by adding 99% acetic acid, for HPLC analysis samples were adjusted to pH 6.0 with 10 M NaOH.

For each experiment a blank sample with 50 mM sodium phosphate buffer (pH 7.5) instead of peptidases, cells, or crude extract was prepared. Biotransformations with resting cells and crude extract were performed in triplicate.

### Analysis

Degradation of DKPs and dihydrouracil was analyzed by RP-HPLC with an Agilent 1200 system (Agilent Technologies, Santa Clara, USA) using a NUCLEODUR Sphinx RP® (4.6 mm ID × 250 mm, 5 μm particle size, Macherey-Nagel Corporation, Düren, Germany) connected with a C_18_ security guard column (3.0 mm ID × 4 mm, Phenomenex, Torrance, USA). The mobile phase consisted of varying ratios of MeOH and 20 mM sodium phosphate buffer (pH 5.5). Analysis of cyclo(Gly-Gly), cyclo(dl-Ala-dl-Ala), cyclo(l-Ala-l-Ala), cyclo(l-Asp-l-Phe), cyclo(Gly-l-Phe), cyclo(l-Asp-l-Asp), cyclo(l-Pro-l-Tyr), cyclo(l-Arg-l-Arg) was performed with validated methods under best separation conditions described by Perzborn et al. (2013). Analysis of cyclo(l-Lys-l-Lys), 1,3-dimethyl-2,5-diketopiperazine and dihydrouracil was performed using 10% MeOH and 90% 20 mM sodium phosphate buffer (pH 5.5) as mobile phase. (*S*)-3-benzyl-1,4-dimethyl-2,5-diketopiperazine and 3-benzyl-3-methyl-2,5-diketopiperazine were analyzed using 45% MeOH and 55% buffer. The flow rate was 0.7 mL/min and the column temperature was set at 20°C (for analysis with 10% MeOH) or at 30°C (for 45% MeOH). The detection wavelength was 210 nm and run time was 15 min for each sample.

## Results

### Taxonomical identification of bacterial strains

The isolates K3, 32A and 40A were identified by amplification and sequencing of the 16S rRNA gene and subsequent comparison with the GenBank® database (Table 1). The 16S rRNA gene of isolate K3 (formerly published as unclassified *Microbacteriaceae* K3) (Dürr et al. 2006) was sequenced for determination of the genus. This strain showed highest identity with strains belonging to the genus *Leifsonia*. The two novel strains 32A and 40A were isolated within this study and were classified by 16S rRNA gene sequencing. Isolate 32A had highest identities with strains of the genus *Paenibacillus.* 40A displayed highest identities with strains belonging to the genus *Microbacterium*. The 16S rRNA gene sequences were submitted to the EMBL Nucleotide Sequence Database, and the strains were deposited at the DSMZ. The strains and the 16S rDNA data are deposited under the DSM and EMBL accession numbers presented in Table 1.

**Table 1:** Results of 16S rRNA gene sequencing

**Isolate**	**Deposited at DSMZ as**	**EMBL accession number**	**Closest relative in GenBank®**	**% identities/bp**
K3	*Leifsonia* sp. DSM 27212	HG322862	*Leifsonia naganoensis* DB103 (NR_043662.1)	100/946
32A	*Paenibacillus* sp. DSM 27214	HG322863	*Paenibacillus chibensis* ZYb3 (FJ432004.1)	99/976
40A	*Microbacterium* sp. DSM 27211	HG322864	*Microbacterium* sp. I_29-J6NFA10A (JQ917793.1)	99/979

*DSM*Z: German Collection of Microorganisms and Cell Cultures, *EMBL accession number:* accession number for the 16S rDNA sequence data at the EMBL nucleotide sequence archive, *number in brackets:* GenBank® accession number, *bp:* sequence length in base pairs.

### Biotransformations with peptidases

Due to the fact that some peptidases are described to cleave DKPs, 15 peptidases were studied for the hydrolysis of different DKPs. None of the tested enzymes was active against any of the substrates (Table 2).

**Table 2:** Results of biotransformation experiments with peptidases and DKPs

**Peptidase**	**Cyclo(Gly-Gly)**	**Cyclo(dl-Ala-dl-Ala)**	**Cyclo(l-Asp-l-Phe)**	**Cyclo(Gly-l-Phe)**	**Cyclo(l-Asp-l-Asp)**	**Cyclo(l-Pro-l-Tyr)**	**Cyclo(l-Arg-l-Arg)**	**Cyclo(l-Lys-l-Lys)**	**( *S *)-3-Benzyl-1,4-dimethyl-2,5-diketopiperazine**	**3-Benzyl-3-methyl-2,5-diketopiperazine**	**1,3-Dimethyl-2,5-diketopiperazine**
Trypsin	-	-	-	-	-	-	-	-	-	-	-
Pepsin	-	-	-	-	-	-	-	n.t.	-	-	-
Papain	-	-	-	-	-	-	-	-	-	-	-
Ficin	-	-	-	n.t.	n.t.	n.t.	n.t.	n.t.	n.t.	n.t.	n.t.
Bromelain	-	-	-	n.t.	n.t.	n.t.	n.t.	n.t.	n.t.	n.t.	n.t.
Chymotrypsin	-	-	-	n.t.	n.t.	n.t.	n.t.	n.t.	n.t.	n.t.	n.t.
Alcalase	-	-	-	n.t.	-	n.t.	-	-	n.t.	n.t.	n.t.
Savinase	-	-	-	n.t.	-	n.t.	-	-	n.t.	n.t.	n.t.
Everlase	-	-	-	n.t.	-	n.t.	-	-	n.t.	n.t.	n.t.
Esperase	-	-	-	n.t.	-	n.t.	-	-	n.t.	n.t.	n.t.
Protex 6L	-	-	-	n.t.	-	n.t.	-	-	n.t.	n.t.	n.t.
Protex 30L	-	-	-	n.t.	-	n.t.	-	-	n.t.	n.t.	n.t.
Protex 40L	-	-	-	n.t.	-	n.t.	-	-	n.t.	n.t.	n.t.
Protex 51FP	-	-	-	n.t.	-	n.t.	-	-	n.t.	n.t.	n.t.
Protex 89L	-	-	-	n.t.	-	n.t.	-	-	n.t.	n.t.	n.t.

“-“ no hydrolysis, n.t. not tested.

### Biotransformations with *Paenibacillus chibensis* (DSM 329) and *Streptomyces flavovirens* (DSM 40062)

**Figure 2:**
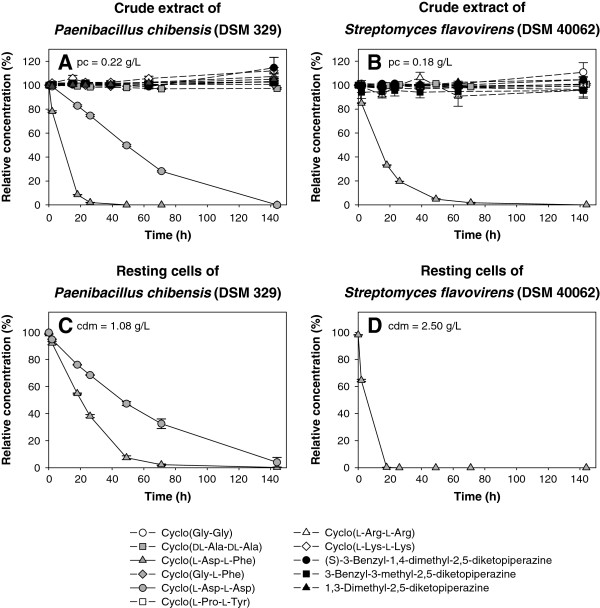
**Substrate spectrum for *Paenibacillus chibensis *(DSM 329) and *Streptomyces flavovirens *(DSM 40062).** Hydrolysis of different DKPs by crude extract of **(A)** DSM 329 and **(B)** DSM 40062, and by resting cells of **(C)** DSM 329 and **(D)** DSM 40062. Each value is the mean of three replicates with error bars representing the standard deviation; pc: protein concentration, cdm: cell dry mass.

For *Paenibacillus chibensis* (DSM 329) and *Streptomyces flavovirens* (DSM 40062) the substrate specificity was investigated with 11 DKPs. The reported hydrolysis of cyclo(l-Asp-l-Phe) (Yokozeki et al. 1990) was shown with crude extract (Figure 2A, B) and resting cells (Figure 2C, D). Moreover, activity towards cyclo(l-Asp-l-Asp) was measured with crude extract and resting cells of DSM 329 (Figure 2A, C). All other tested DKPs were not cleaved by both strains.

**Figure 3:**
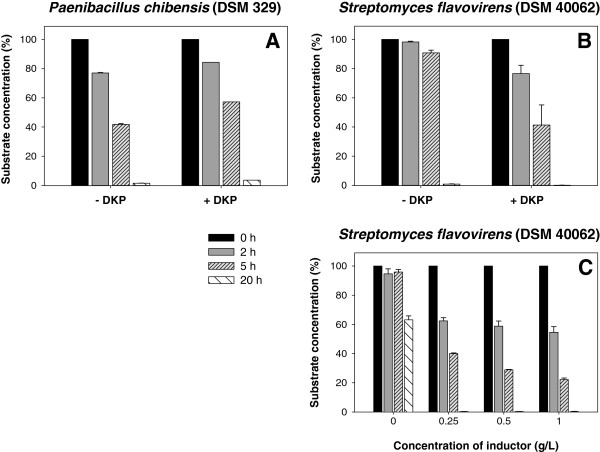
**Enzyme induction after cultivation with DKPs for *Paenibacillus chibensis *(DSM 329) and *Streptomyces flavovirens *(DSM 40062).** Hydrolysis of cyclo(l-Asp-l-Phe) by resting cells of **(A)** DSM 329 and **(B)** DSM 40062 without induction (− DKP) and with induction (+ DKP) during cultivation. **(C)** Hydrolysis of cyclo(l-Asp-l-Phe) by crude extract of DSM 40062 after cultivation with different inductor concentrations. Each value is the mean of three replicates with error bars representing the standard deviation.

In addition, the induction of enzyme activity was examined after cultivation with cyclo(Gly-Gly), cyclo(dl-Ala-dl-Ala) and cyclo(l-Asp-l-Phe) (Figure 3A, B).

No difference in the hydrolysis rate of the substrate cyclo(l-Asp-l-Phe) was detected for DSM 329 (Figure 3A). In contrast, for DSM 40062 an enhanced activity towards cyclo(l-Asp-l-Phe) was determined after induction with DKPs during the cultivation (Figure 3B). The enzyme activity increased with raising inductor concentrations (0.25 till 1 g/L cyclo(l-Asp-l-Phe)), but the enzyme was also active towards this DKP without induction (Figure 3C).

### Biotransformations with strains exhibiting cyclic amidase activity

**Figure 4:**
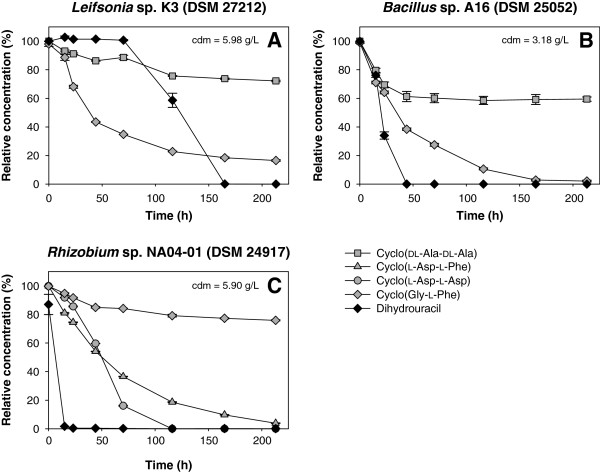
**Degradation of different DKPs by (A) *Leifsonia *sp. K3 (DSM 27212), (B) *Bacillus *sp. A16 (DSM 25052) and (C) *Rhizobium *sp. NA04-01 (DSM 24917).** Biotransformations were done with resting cells of the three strains. Each value is the mean of three replicates with error bars representing the standard deviation; cdm: cell dry mass.

34 strains hydrolyzing cyclic amides (e.g., hydantoin or dihydrouracil) of the in-house strain collection were tested for hydrolysis of cyclo (Gly-Gly), cyclo (dl-Ala-dl-Ala), cyclo (l-Asp-l-Phe), cyclo (Gly-l-Phe), cyclo (l-Asp-l-Asp), cyclo (l-Pro-l-Tyr), (*S*)-3-benzyl-1,4-dimethyl-2,5-diketopiperazine, 3-benzyl-3-methyl-2,5-diketopiperazine and 1,3-dimethyl-2,5-diketopiperazine. Dihydrouracil was used as positive control and was cleaved by all 34 strains. Moreover, three of these strains were identified for degradation of DKPs. *Leifsonia* sp. K3 (DSM 27212) and *Bacillus* sp. A16 (DSM 25052) showed activity towards cyclo(dl-Ala-dl-Ala) and cyclo(Gly-l-Phe) (Figure 4A, B). *Rhizobium* sp. NA04-01 (DSM 24917) cleaved cyclo(l-Asp-l-Phe), cyclo(l-Asp-l-Asp) and cyclo(Gly-l-Phe) (Figure 4C). All other strains showed no significant activity towards the investigated DKPs.

### Biotransformations with novel bacterial isolates

**Figure 5:**
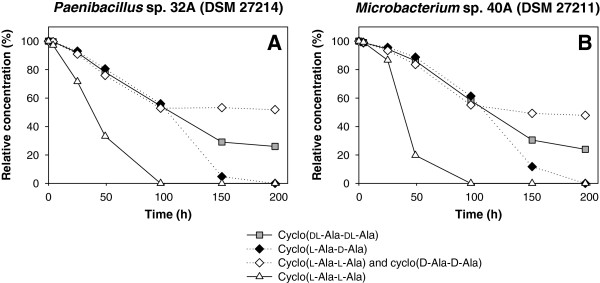
**Enantioselective degradation of the enantiomers of cyclo(dl-Ala-dl-Ala) by (A) *Paenibacillus *sp. 32A (DSM 27214) and (B) *Microbacterium *sp. 40A (DSM 27211).** Resting cells of both strains were used and OD_600nm_ of the biotransformation samples was 4.75.

The bacterial strains *Paenibacillus* sp. 32A (DSM 27214) and *Microbacterium* sp. 40A (DSM 27211) were isolated and classified (Table 1). Both isolates were identified for degradation of cyclo(dl-Ala-dl-Ala) (Figure 5). However, only 75% of this racemic DKP was cleaved by these strains. As the meso compound cyclo(l-Ala-d-Ala) can be separated from the enantiomeric pair composed of cyclo(l-Ala-l-Ala) and cyclo(d-Ala-d-Ala) by HPLC (Perzborn et al. 2013), the enantioselective degradation of cyclo(dl-Ala-dl-Ala) by resting cells of DSM 27214 and DSM 27211 was investigated (Figure 5). Both strains degraded 100% of cyclo(l-Ala-d-Ala) and only 50% of the enantiomeric pair cyclo(l-Ala-l-Ala) and cyclo(d-Ala-d-Ala). Besides the racemic cyclo(dl-Ala-dl-Ala), the enantiomer cyclo(l-Ala-l-Ala) was used as substrate and 100% degradation was detected. Therefore, it can be concluded that the two novel isolated bacteria are not able to cleave the enantiomer cyclo(d-Ala-d-Ala). Thus, with DSM 27214 and DSM 27211 the first enantioselective degradation of cyclo(dl-Ala-dl-Ala) was shown. DSM 27214 and DSM 27211 were not active towards the dihydropyrimidine dihydrouracil.

## Discussion

Due to the lack of consistent and detailed information about the degradation of DKPs, four approaches in order to identify and characterize novel biocatalysts for the cleavage of DKPs were pursued.

### Stability of the peptide bond in DKPs towards peptidases

The cleavage of the peptide bond in DKPs by various peptidases was studied. Therefore, peptidases described for DKP hydrolysis, as well as non-studied peptidases and DKPs were investigated. The reported stability of cyclo(Gly-Gly) towards trypsin, pepsin (Waldschmidt-Leitz and Schäffner 1925), (Ishiyama 1933) and papain (Waldschmidt-Leitz and Schäffner 1925), and of cyclo(Asp-Asp) towards pepsin (Matsui 1933), (Ishiyama 1933) was confirmed. In contrast, the described hydrolysis of cyclo(Asp-Asp) by trypsin (Matsui 1933), (Ishiyama 1933), (Shibata and Tazawa 1936) and papain (Shibata and Tazawa 1936), and of cyclo(Arg-Arg) by pepsin (Shibata and Tazawa 1936) could not be confirmed. The divergence in activity could be reasoned by different enzyme preparations and used analytical methods. The enzyme purity was probably not as good as in the preparation used in this study, e.g., the trypsin used by Shibata and Tazawa (1936) was only enriched by adsorption on aluminium hydroxide according to Waldschmidt-Leitz and Schäffner (1925). Thus, the measured activities may result from unknown enzymes and not from the named peptidases. Matsui (1933), Ishiyama (1933) and Shibata and Tazawa (1936) analyzed the DKP hydrolysis by detection of changes in the pH-value caused by the released carboxyl group. Additionally, Shibata and Tazawa (1936) detected the formation of free amino groups according to Van Slyke (1911). These methods are not as specific and exact as the validated HPLC methods used in this study to measure decreasing substrate concentration. In addition, there may be specific reasons, such as the use of different enantiomers. Shibata and Tazawa (1936) described the hydrolysis of cyclo(d-Arg-d-Arg). In contrast, in this study the hydrolysis of the corresponding l-enantiomer was examined.

Investigations with further peptidases, e.g., bromelain, ficin and various subtilisins, and additional DKPs, e.g., cyclo(dl-Ala-dl-Ala), cyclo(l-Asp-l-Phe) and cyclo(l-Pro-l-Tyr) did not result in positive reactions. No cleavage of the peptide bond in DKPs could be detected by using 15 peptidases and eleven DKPs. This indicates extraordinary peptidase stability of the peptide bond in cyclic dipeptides. This may be an advantage for the use of DKPs as peptide drugs in some indications, such as cancer or infectious diseases. It was shown that cyclo(His-Phe) reduced the viability of cervical cancer cells (McCleland et al. 2004). The DKP bicyclomycin inhibited the growth of various gram-negative bacteria (Miyamura et al. 1972).

### Substrate specificity and enzyme induction with *Paenibacillus chibensis* (DSM 329) and *Streptomyces flavovirens* (DSM 40062)

In our studies we could confirm the reported hydrolysis of cyclo(l-Asp-l-Phe) by *Paenibacillus chibensis* (DSM 329) and *Streptomyces flavovirens* (DSM 40062) (Yokozeki et al. 1990). Moreover, DSM 329 was active against cyclo(l-Asp-l-Asp), but no degradation of nine other tested DKPs was measured. DSM 40062 showed no activity towards any of the ten investigated DKPs. These results indicate high substrate specificity for the enzymes of both strains. High substrate specificity is also reported for the cyclo(Gly-Gly) hydrolase of *Bacillus* sp. No. 106, which was active towards one of 32 tested DKPs (Muro et al. 1985). In contrast, a relatively wide substrate spectrum was shown for *Arthrobacter* sp. 1-3-1 and coryneform rod bacterium T-1-3-Y with activity towards 12 DKPs (Kanzaki et al. 1997).

The cleavage of two DKPs with an aspartyl residue (cyclo(l-Asp-l-Phe), cyclo(l-Asp-l-Asp)) by DSM 329 was demonstrated, showing a preference for DKPs with this residue. Another substrate with a phenylalanine residue (cyclo(l-Gly-l-Phe)) was not attacked, indicating that rather the acidic residue of cyclo(l-Asp-l-Phe) is responsible for enzyme activity than the aromatic residue. It is important to note that the activities were detected using resting cells and crude extracts. Thus, further investigations are needed to clarify, if both DKPs are cleaved by the same or by two distinct enzymes.

For both strains the induction of enzyme activity by DKPs was investigated. After cultivation with and without DKPs, no difference in enzyme activity was measured for DSM 329. In contrast, the degradation of cyclo(l-Asp-l-Phe) by DSM 40062 was shown to be inducible by this DKP during cultivation. The activity increased with raising inductor concentration, but the enzyme was also active without previous induction. Kanzaki et al. (1997) observed comparable results for *Arthrobacter* sp. 1-3-1 and coryneform rod bacterium T-1-3-Y. With these strains the DKP hydrolyzing activity was also induced in presence of DKPs in the cultivation medium.

### Degradation of DKPs with strains hydrolyzing other cyclic amides (hydantoins and dihydropyrimidines)

Strains with known cyclic amidase activity, cleaving hydantoins and dihydropyrimidines (Dürr et al. 2006, 2008; Engel et al. 2012) were examined regarding the degradation of the structurally similar DKPs (see Figure 1). The cleavage of the dihydropyrimidine dihydrouracil, used as positive control, could be shown for all 34 tested strains. For *Rhizobium* sp. NA04-01 (DSM 24917) and for two *E. coli* strains with recombinant hydantoinases of *Ochrobactrum* sp. G21 (with pJOE5702.1) and of *Delftia* sp. I24 (with pJOE5704.1) cleavage of dihydrouracil was shown for the first time.

Furthermore, three of the tested bacteria were newly identified for the degradation of DKPs. *Leifsonia* sp. K3 (DSM 27212) (formerly unclassified *Microbacteriaceae*) described for the hydrolysis of 5-benzylhydantoin and dihydrouracil (Dürr et al. 2006) was active towards cyclo(dl-Ala-dl-Ala) and cyclo(Gly-l-Phe). Thus, two substrates with benzyl substituent, 5-benzylhydantoin and cyclo(Gly-l-Phe), are degraded. Cleavage of cyclo(dl-Ala-dl-Ala) and cyclo(Gly-l-Phe) was detected with *Bacillus* sp. A16 (DSM 25052). A wide substrate spectrum with activity towards hydantoin, 5-*tert*-butylhydantoin, 5-(3-indolylmethyl)-hydantoin (Dürr 2007), 5-benzylhydantoin, dihydrouracil (Dürr et al. 2006), 6-phenyldihydrouracil and *p*-chloro-6-phenyldihydrouracil (Engel et al. 2012) was reported for this strain. *Rhizobium* sp. NA04-01 (DSM 24917) degraded cyclo(l-Asp-l-Phe), cyclo(l-Asp-l-Asp) and cyclo(Gly-l-Phe). The substrate spectrum indicates a preference for DKPs with either phenylalanine or aspartic acid. This strain is known for the hydrolysis of 6-phenyldihydrouracil and *p*-chloro-6-phenyldihydrouracil (Engel et al. 2012). In general, DSM 27212 and DSM 25052 are able to hydrolyze at least one hydantoin, one dihydropyrimidine and two DKPs. DSM 24917 cleaves three dihydropyrimidines and three DKPs; activity towards hydantoins has not been investigated, yet. To our knowledge, these three bacteria are the first strains identified for the degradation of DKPs and dihydropyrimidines. Former studies reported hydrolysis of cyclo(Gly-Gly), but no activity towards the dihydropyrimidine dihydrouracil with the cyclo(Gly-Gly) hydrolases of *Bacillus* sp. No. 106 was detected (Muro et al. 1985).

The cleavage of hydantoins, dihydropyrimidines and DKPs were examined with resting cells. Hence, it remains unclear if the same enzymes are responsible for the degradation of these different molecules. Two *E. coli* strains with recombinant d-hydantoinases were tested for DKP hydrolysis to discuss this question. The recombinant enzymes of *Ochrobactrum* sp. G21 and *Delftia* sp. I24 are known for the hydrolysis of 5-benzylhydantoin, 5-(3-indolylmethyl)-hydantoin (Dürr et al. 2008), 6-phenyldihydrouracil and *p*-chloro-6-phenyldihydrouracil (Engel et al. 2012). We identified hydrolysis of the dihydropyrimidine dihydrouracil for both recombinant enzymes, but no activity towards one of the nine tested DKPs could be detected. Thus, it needs further investigations to clarify, if this enzyme class is able to degrade DKPs.

### Identification of novel isolates with enantioselective DKP cleavage activity

Two novel isolates were identified for the degradation of cyclo(dl-Ala-dl-Ala). These strains were classified as *Paenibacillus* sp. 32A (DSM 27214) and *Microbacterium* sp. 40A (DSM 27211) by 16S rRNA gene sequence analysis and alignment. The enantioselectivity towards this racemic substrate was investigated for both strains: the enantiomers cyclo(l-Ala-l-Ala) and cyclo(l-Ala-d-Ala) were completely degraded, whereas no cleavage of cyclo(d-Ala-d-Ala) was detected. Thus, enantioselectivity towards substrates containing at least one l-Ala was identified. Enantioselective DKP hydrolysis was studied by Kanzaki et al. (1997, 2000) for *Arthrobacter* sp. 1-3-1, coryneform rod bacterium T-1-3-Y and *Agrobacterium radiobacter* NM 5-3: *Arthrobacte*r sp. 1-3-1 preferred cyclo(Gly-l-Ala) to cyclo(Gly-d-Ala), coryneform rod bacterium T-1-3-Y was more active towards cyclo(Gly-l-Leu) than towards the corresponding d-enantiomer, and *Agrobacterium radiobacter* NM 5-3 showed no significant preference for one of the enantiomers of cyclo(Gly-dl-Ala) or cyclo(Gly-dl-Leu) (Kanzaki et al. 2000). None of these strains was strictly enantioselective towards any DKP. Thus, with the novel isolates *Paenibacillus* sp. 32A (DSM 27214) and *Microbacterium* sp. 40A (DSM 27211) the first enantioselective degradation of a racemic DKP could be demonstrated. Therefore, these two bacteria may be of particular interest for further investigations concerning the characterization of DKP degrading enzymes. Moreover, both strains could be used for industrial applications, e.g., production of enantiopure DKPs or dipeptides.

Furthermore, these two strains were tested for the hydrolysis of dihydrouracil in comparison to the strains of the in-house strain collection. However, DSM 27214 and DSM 27211 were not active towards dihydrouracil, indicating that the detected DKP cleavage probably does not rely on dihydropyrimidinases.

### General aspects of DKP degradation

Altogether, five bacteria were newly identified for degradation of four DKPs. None of the investigated peptidases showed activity towards DKPs. Thus, it remains unclear which enzymes are responsible for the hydrolysis of DKPs. Muro et al. (1985) concluded, that the cyclo(Gly-Gly) hydrolase of *Bacillus* sp. No. 106 is a novel enzyme which is different in substrate specificity compared to peptidases, because no hydrolysis of milk casein, hemoglobin, Gly-Gly, barbital, barbituric acid and dihydrouracil could be detected.

*Paenibacillus chibensis* (DSM 329), *Leifsonia* sp. K3 (DSM 27212), *Bacillus* sp. A16 (DSM 25052) and *Rhizobium* sp. NA04-01 (DSM 24917) were able to cleave more than one DKP. It has to be investigated, if the same or distinct enzymes are responsible for degradation of the different substrates.

To the best of our knowledge, this is the first report describing DKP degradation with bacteria of the genera *Microbacterium*, *Leifsonia* and *Rhizobium*. Further strains of the genera *Bacillus* and *Paenibacillus* were identified in addition to the known DKP hydrolyzing strains *Bacillus* sp. No. 106 (Muro et al. 1985) and *Paenibacillus chibensis* (DSM 329) (Yokozeki et al. 1990).

The cleavage of cyclo(l-Asp-l-Phe), cyclo(Gly-l-Phe), cyclo(dl-Ala-dl-Ala) and cyclo(l-Asp-l-Asp) was shown. Hydrolysis of cyclo(l-Asp-l-Phe) is described for eleven strains (Yokozeki et al. 1990), e.g., *Paenibacillus chibensis* (DSM 329) and *Streptomyces flavovirens* (DSM 40062) which were also investigated within this study. Hydrolysis of cyclo(Gly-l-Phe) is known for *Arthrobacter* sp. 1-3-1 und coryneform rod bacterium T-1-3-Y (Kanzaki et al. 1997), while degradation of cyclo(dl-Ala-dl-Ala) was discovered for the first time by identification of *Leifsonia* sp. K3 (DSM 27212), *Bacillus* sp. A16 (DSM 25052), *Paenibacillus* sp. 32A (DSM 27214) and *Microbacterium* sp. 40A (DSM 27211) as biocatalysts for this substrate. The first microbial hydrolysis of cyclo(l-Asp-l-Asp) was identified by using *Paenibacillus chibensis* (DSM 329) and *Rhizobium* sp. NA04-01 (DSM 24917). None of all the examined strains was able to degrade the widespread cyclo(l-Pro-l-Tyr), which is synthesized by bacteria like *Pseudomonas aeruginosa* (Holden et al. 1999) and *Actinomyces* sp. (Arunrattiyakorn et al. 2006), fungi e.g., *Alternaria alternata* (Stierle et al. 1988), and sponges, such as *Jaspis digonoxea* (Rudi et al. 1994). Furthermore, no strain could be identified for cleavage of the simplest DKP cyclo(Gly-Gly), alkaline DKPs cyclo(l-Arg-l-Arg), cyclo(l-Lys-l-Lys) and three DKPs containing non-proteinogenic amino acids (*S*)-3-benzyl-1,4-dimethyl-2,5-diketopiperazine, 3-benzyl-3-methyl-2,5-diketopiperazine and 1,3-dimethyl-2,5-diketopiperazine.

## Competing interests

The authors declare that they have no competing interests.
